# *NIPTeR*: an R package for fast and accurate trisomy prediction in non-invasive prenatal testing

**DOI:** 10.1186/s12859-018-2557-8

**Published:** 2018-12-17

**Authors:** Lennart F. Johansson, Hendrik A. de Weerd, Eddy N. de Boer, Freerk van Dijk, Gerard J. te Meerman, Rolf H. Sijmons, Birgit Sikkema-Raddatz, Morris A. Swertz

**Affiliations:** 1 Department of Genetics, University of Groningen, University Medical Center Groningen, Groningen, The Netherlands; 2 Genomics Coordination Center, University of Groningen, University Medical Center Groningen, Groningen, The Netherlands; 3 School of Bioscience, Systems biology research center, University of Skövde, Skövde, Sweden

**Keywords:** NIPT, Trisomy prediction, Next-generation sequencing

## Abstract

**Background:**

Various algorithms have been developed to predict fetal trisomies using cell-free DNA in non-invasive prenatal testing (NIPT). As basis for prediction, a control group of non-trisomy samples is needed. Prediction accuracy is dependent on the characteristics of this group and can be improved by reducing variability between samples and by ensuring the control group is representative for the sample analyzed.

**Results:**

*NIPTeR* is an open-source R Package that enables fast NIPT analysis and simple but flexible workflow creation, including variation reduction, trisomy prediction algorithms and quality control. This broad range of functions allows users to account for variability in NIPT data, calculate control group statistics and predict the presence of trisomies.

**Conclusion:**

*NIPTeR* supports laboratories processing next-generation sequencing data for NIPT in assessing data quality and determining whether a fetal trisomy is present. *NIPTeR* is available under the GNU LGPL v3 license and can be freely downloaded from https://github.com/molgenis/NIPTeR or CRAN.

**Electronic supplementary material:**

The online version of this article (10.1186/s12859-018-2557-8) contains supplementary material, which is available to authorized users.

## Background

Non-invasive prenatal testing (NIPT) is rapidly becoming the new standard in prenatal screening for fetal aneuploidy [1]. In NIPT, cell-free DNA from the pregnant woman’s blood plasma, which consists of both maternal and fetal DNA fragments, is analysed. Next to SNP-based methods [2], low-coverage whole genome next-generation sequencing (NGS) is often used [3, 4], and various algorithms, software programs and packages have been developed to analyse this type of data [5–9]. In literature, many methods have been described that depend on a statistical comparison between a sample of interest and a reference set of non-trisomy control samples [3, 4, 10, 11]. The *RAPIDR* and *DASAF* R packages, for instance, have been described [12, 13] and they made several of these algorithms available, including GC-correction, the standard Z-score and the Normalized Chromosome Value (NCV), to create an analysis workflow in R. However, those packages lack features like chi-squared-based variation reduction (χ^2^VR), regression-based Z-score (RBZ) and Match QC. These are all algorithms that we have extensively discussed before [11]. In short, χ^2^VR detects chromosomal regions that have a higher variability than expected by chance and reduces their weight so that, after correction, they have less impact on the fraction of reads mapped to the different chromosomes. The RBZ is an alternative Z-score calculation based on stepwise regression with forward selection. In the RBZ positive or negative correlation between chromosomal fractions is used to predict the number of reads to map onto the chromosome of interest if no trisomy is present. The Match QC score is a sum-of-squares-based approach to compare chromosomal fractions between the test sample and controls, and it provides a measure by which to determine whether a control group is representative for a specific sample. Here we report *NIPTeR*, an R package that provides fast NIPT analysis for research and diagnostics and provides users with multiple methods for variation reduction, prediction and quality control based upon comparison of a sample with a set of negative control samples.

## Implementation

**Fig. 1:**
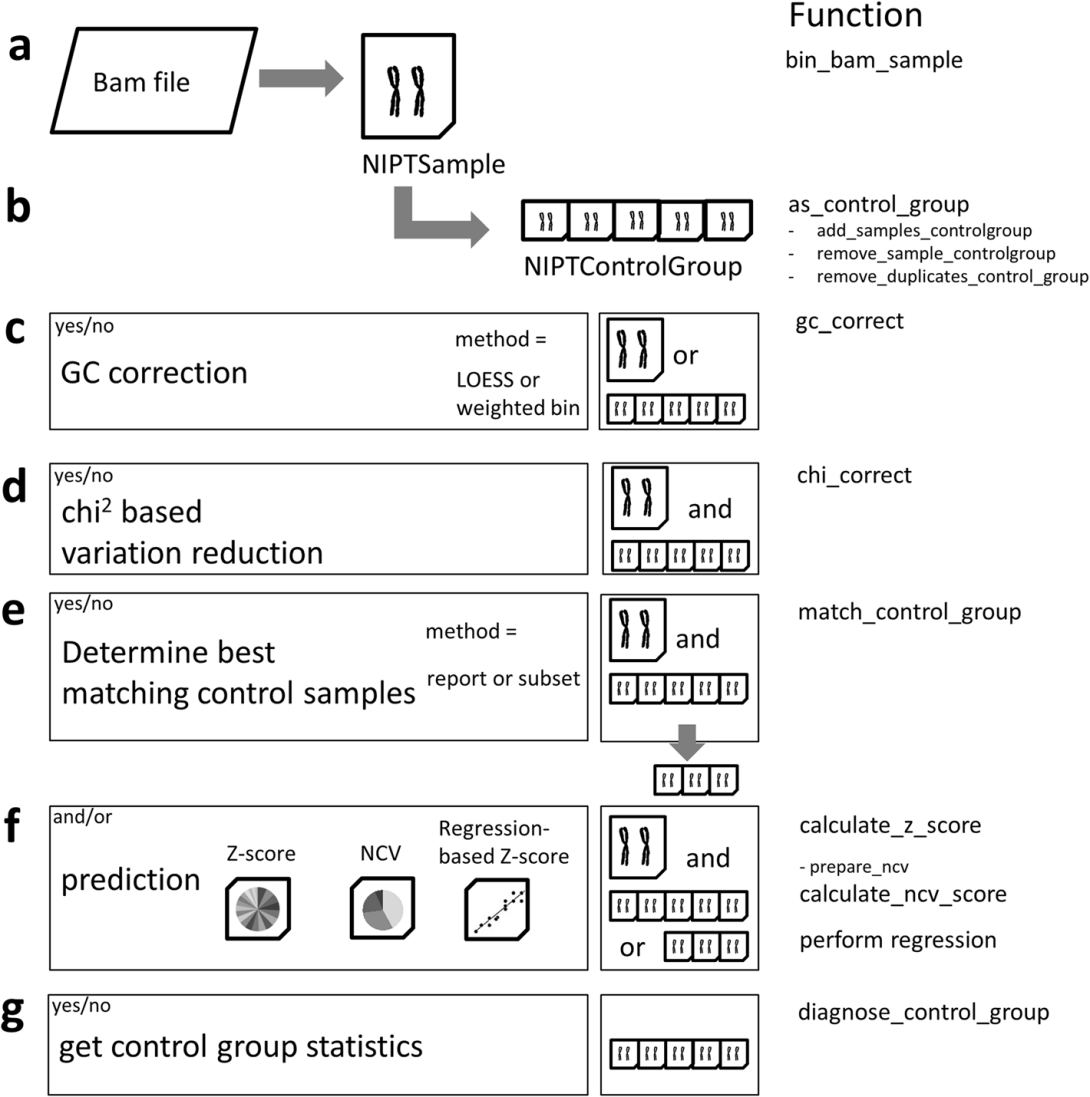
Workflow and functions of *NIPTeR*. **a** A BAM file is transformed into an NIPTSample object; **b** a series of NIPTSample objects can then be transformed into an NIPTControlGroup object; **c** optional LOESS or weighted bin GC correction; **d** optional chi-squared-based variation reduction; **e** optional comparison of NIPTSample and NIPTControlGroup and possible selection of a subset that best-matches the control group samples; **f** three different prediction methods: Z-score, normalized chromosome value or regression-based Z-score; **g** optional check of control group statistics

*NIPTeR* users can create different workflows for variation reduction and aneuploidy prediction using thirteen functions as building blocks (Fig. 1). A stepwise practical example for using these building blocks is presented as a case report in Additional file 1.

*NIPTeR* analysis uses two core objects. The first object is *NIPTSample*, which contains the counts of aligned sequence reads in 50,000 bp bins for a specific sample. The second object is *NIPTControlGroup*, which contains a series of NIPTSamples for comparison. Users generate *NIPTSample* using the function *bin_bam_sample*, which needs a BAM file [14] as input. The user can optionally select to count reads mapped to the forward and reverse strands separately, so that they can each be used as a separate predictor. The *as_control_group* function converts a series of *NIPTSample* objects into a *NIPTControlGroup*. Within *NIPTeR*, users can manage an existing *NIPTControlGroup* using the *add_samples_controlgroup*, *remove_sample_controlgroup* and *remove_duplicates_controlgroup* functions.

Both *NIPTSample* and *NIPTControlGroup* can undergo one or more variation reduction steps to adjust the bin read counts, either using the *gc_correct* function for weighted bin GC correction [10] or LOESS GC correction [15] or the *chi_correct* function for χ^2^VR. Each *NIPTSample* object shows the correction status for the autosomes and the sex chromosomes separately and indicates which variation reduction methods have been performed (or that they are ‘uncorrected’). χ^2^VR can be applied to uncorrected or GC-corrected samples, and makes use of a *NIPTSample* and a *NIPTControlGroup* having an identical correction status.

Using the fractions of reads mapped to the different chromosomes, trisomy prediction can be generated for a given *NIPTSample* based on the *NIPTControlGroup* using three different prediction algorithms: (1) *calculate_z_score*, which uses a standard Z-score [3]; (2) *calculate_ncv_score*, which uses an NCV [4]; and (3) *perform_regression*, which uses RBZ. All three trisomy prediction functions use *NIPTControlGroup* to calculate the expected fraction of reads on the chromosome of interest. For NCV, this calculation is done in a separate function, *prepare_ncv*, because the calculation is time-intensive and only has to be performed once for each *NIPTControlGroup*. The prediction functions then compare the observed fraction of reads of the chromosome of interest in the *NIPTSample* with the expected fraction. In NCV and RBZ calculations, users have the option of excluding selected chromosomes as predictors. Since chromosomes 13, 18 and 21 are the most likely candidates for a trisomy, these are excluded by default, but users do have the option of including them. The functions *prepare_ncv* and *perform_regression* provide users the option of using a train and test set to prevent over-fitting the models they create.

In addition to providing Z-scores, the functions also produce control group statistics. The function *match_control_group* provides a Match QC score, a calculation that shows how well the sample fits within the control group based on the fraction of reads mapped to the different chromosomes, a measure that can be shown in a report. Alternately, users can select a subset of best-matching control samples as a sample-specific control group using the arguments mode = “report” or “subset”. When a sample has an anomalously high Match QC score, the control samples being used are not suitable as a control group for the sample being analyzed. A second quality control function, *diagnose_control_group*, calculates Z-scores for all samples and chromosomes in a *NIPTControlGroup* as well as the mean, standard deviation and Shapiro-Wilk test of those Z-scores. This information can be used to curate the control group as explained in detail in Additional file 1.

## Results

### Workflow

All these *NIPTeR* building blocks can be combined into an analysis workflow. For example, the *NIPTeR* workflow for the Fan & Quake analysis [10], using a weighted bin GC correction and a standard Z-score prediction for trisomy 21, and given a GC-corrected control group is:

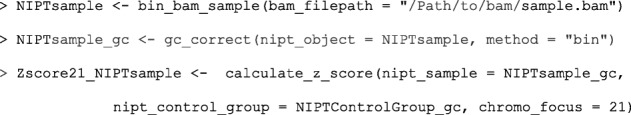



In addition, control group statistics and the match control of the sample to the control group can be performed:

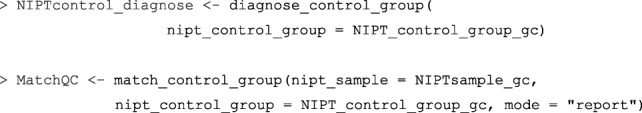



### Prediction and control group statistics

The output formats of the *calculate_z_score* and *calculate_ncv_score* functions are similar. An example result of the main output reads:

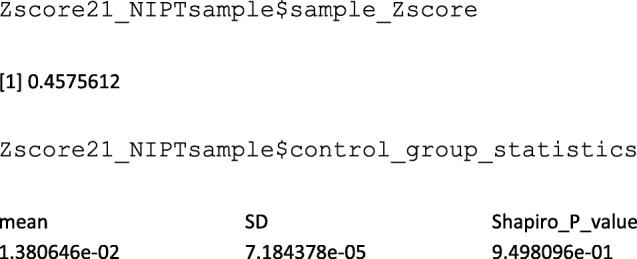



Here, the Z-score is 0.45, which falls within the − 3 to 3 range and leads to the conclusion that this sample does not have a trisomy 21. The control_group_statistics show the mean fraction of sequence reads mapping to chromosome 21 and the standard deviation (SD) of the fractions between the control samples. The Shapiro_P_value tests for control group normality, and control groups with a value above 0.05 can be considered to be normally distributed.

The output of *perform_regression* is slightly different and gives four predictions based on different models when set to the default setting:

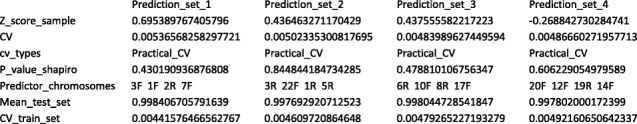



Here, in addition to the RBZ, the coefficient of variation (CV) of the test set is given as a measure of control group variability. The type of CV is given as well, in which “Practical CV” is the true CV. If there is a risk of over-fitting the model on the control set, a theoretical CV is used. In addition to the Shapiro *P* value, *perform_regression* reports the mean of the test set (which should be close to one) and the CV of the training set (based on which the chromosomes used to create the prediction model are selected), where reads mapped to the forward and reverse strands are used as separate entities.

### Quality control

Using the *diagnose_control_group* function, control samples that have outliers that could hamper prediction can be detected.

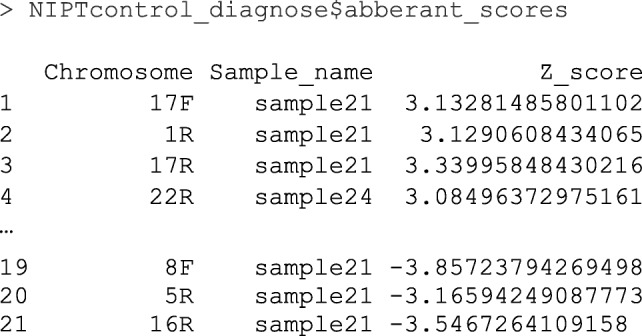



This example shows that, for many chromosomes in sample 21 one or both of the strands have a Z-score higher than 3. This means that there is more variability in this sample than expected, pointing to a low quality sample. As explained in more detail in Additional file 1, we recommend that users remove samples that have more than one aberrant score (Z-score outside the − 3 to 3 range) from the control group.

When looking at the individual Match QC scores of the GC corrected *NIPTSample* compared to the GC corrected *NIPTControlGroup*, the list of sum of squares of differences in chromosomal fractions of the test sample compared to each control sample is shown:

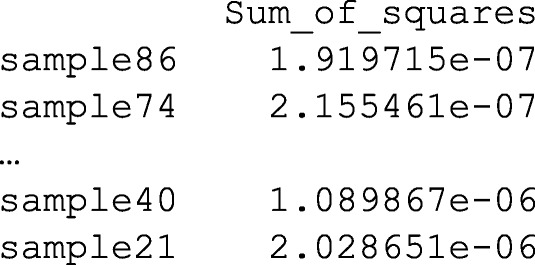



In general, the lower the sum of squares, the more representative a control sample is for the test sample. The average of all sum of squares for an *NIPTSample* is the Match QC score. A Match QC score for a specific sample that falls outside 3 SD of the control group Match QC, indicates that the control group is not suitable for analysis of the sample.

Further examples and results can be found in the *NIPTeR* package vignette [16] and the case report provided in Additional file 1. A demonstration of the *NIPTeR* GC-correction methods is given in Additional file 2 and a comparison of *NIPTeR* results with manual calculations is available for the χ2VR in Additional file 3 and for the prediction methods and Match QC score in Additional file 4.

The *NIPTeR* package requires R 3.1.0 or higher, the stats and sets packages as available on CRAN, and the RSamtools and S4Vectors Bioconductor packages.

### Performance

*NIPTeR* performance was tested on three different machines and operating systems (Additional file 5). Given a pre-processed control group of 100 samples, one sample was processed in 3 to 4 min (on average), including both GC correction and χ^2^VR and using the Z-score and RBZ as prediction algorithms for chromosomes 13, 18 and 21. NCV analysis was performed in an additional 1 to 6 min using a maximum number of 6 to 9 chromosomes as denominator.

## Conclusion

*NIPTeR* allows for fast NIPT analysis and flexible workflow creation and includes variation correction and prediction algorithms as well as QC control. Algorithms used in *NIPTeR* are validated as described in Johansson and de Boer et al. (2017) [11]. *NIPTeR* is available under the GNU GPL open source license and can be freely downloaded from https://github.com/molgenis/NIPTeR or CRAN.

## Availability and requirements

** Project name:** NIPTeR.

**Project home page:** https://CRAN.R-project.org/package=NIPTeR

**Source page:** https://github.com/molgenis/NIPTeR

**Operating system(s)**: Linux, MacOS, Windows.

**Programming language:** R.

**Other requirements:** R (3.1.0 or higher), RSamtools, sets, stats, S4Vectors.

**Licence:** GNU Lesser General Public License v3.0.

**Any restrictions to use by non-academics:** none.

## Additional files

Additional file 1: A step by step case report describing how to create a control group and how to analyse a sample using *NIPTeR*. (DOCX 53 kb)

Additional file 2: Supplemental information showing the functionality of *NIPTeR* bin and LOESS GC correction. (DOCX 682 kb)

Additional file 3: Supplemental information comparing the *NIPTeR* chi-squared based variation reduction calculation with a manual calculation. (XLSX 67 kb)

Additional file 4: Supplemental information comparing the manual calculations for the standard Z-score, Normalized Chromosome Value, Regression-based Z-score and the Match QC with *NIPTeR* calculations. (XLSX 426 kb)

Additional file 5: Supplemental information showing run times per *NIPTeR* function on Linux, MacIntosh and Windows platforms. (XLSX 29 kb)

## Abbreviations

CV: Coefficient of variation
NCV: Normalized Chromosome Value
NIPT: Non-invasive prenatal testing
RBZ: Regression based Z-score
χ^2^VR: Chi-squared based variation reduction
