# Supplementary material for: NIPTeR: an R package for fast and accurate trisomy prediction in non-invasive prenatal testing
Source: BMC Bioinformatics. 2018 Dec 17;19:531. doi: 10.1186/s12859-018-2557-8 (PMC6296037; doi:10.1186/s12859-018-2557-8)
Supplement: Supplementary file 1 — A step by step case report describing how to create a control group and how to analyse a sample using NIPTeR. (DOCX 53 kb) [file 12859_2018_2557_MOESM1_ESM.docx]

*NIPTeR* analysis case report: trisomy 21

Contents

[Introduction 2](#_Toc531332746)

[Control group creation 2](#_Toc531332747)

[Preliminary control group creation 2](#_Toc531332748)

[Diagnose preliminary control group 3](#_Toc531332749)

[Create corrected cleaned control group 3](#_Toc531332750)

[Diagnose corrected cleaned control group 4](#_Toc531332751)

[Create second corrected cleaned control group 4](#_Toc531332752)

[Diagnose second corrected cleaned control group 5](#_Toc531332753)

[Create third corrected cleaned control group 6](#_Toc531332754)

[Diagnose third corrected cleaned control group 6](#_Toc531332755)

[Save cleaned control group for later use 7](#_Toc531332756)

[Set control group Match QC threshold. 7](#_Toc531332757)

[Sample analysis 9](#_Toc531332758)

[GC correction and chi-squared-based variation reduction (χ^2^VR) 9](#_Toc531332759)

[Sample Match QC 10](#_Toc531332760)

[Trisomy prediction 10](#_Toc531332761)

[Standard Z-score 10](#_Toc531332762)

[Normalized Chromosome Value (NCV) 11](#_Toc531332763)

[Regression-based Z-score (RBZ) 12](#_Toc531332764)

[Conclusion 14](#_Toc531332765)

[References 14](#_Toc531332766)

# Introduction

This case report describes how to use the *NIPTeR* R package ([https://CRAN.R-project.org/package=NIPTeR](https://cran.r-project.org/package=NIPTeR)) to analyze Non-Invasive Prenatal Testing (NIPT) data produced using low-coverage whole genome sequencing. We describe how to create a control group, set quality thresholds, reduce variability between samples and predict if a trisomy is present or not.

Note that each laboratory needs to create their own control group based on their laboratory, sequencing and bioinformatics procedures. For this reason the control group provided in this case report can‘t be used for analysis of samples other than the case report sample.

# Control group creation

## Preliminary control group creation

A preliminary control group was created using 100 non-trisomy samples using a Hiseq 2500 platform (Illumina, San Diego, CA, USA), produced as described in Johansson and de Boer et al (2017). To enable calculation of the regression-based Z-score (RBZ) later in this protocol, we set the ‘separate strands’ option to ‘True’ to calculate the fraction of reads present on the forward and reverse strands of each chromosome separately. To create the control group we first enter the path to the BAM files of the control group samples. The control group object can be saved as an .rds file to enable reloading in later instances. In general, to create a preliminary control group, we recommend users to start with all samples (at least 30, but ideally 100 or more) that have undergone the same sample preparation and sequencing procedure as the samples to be analyzed, excluding known trisomy samples. In this step, using the bin_bam_sample function, read counts of 50,000 bp bins are made for all chromosomes. These bin counts form the basis for further analysis, variation reduction and prediction. In *NIPTeR* the bin size is fixed to 50,000 bp.

> ControlGroupDir <- "/PATH/TO/FOLDER/control_bams/"

> bam_filepaths <- list.files(path= ControlGroupDir,

pattern = ".bam", full.names = T )

> NIPT_control_group_separate <- as_control_group(nipt_samples =

lapply(X = bam_filepaths, bin_bam_sample, do_sort = F, separate_strands = T))

> saveRDS(object = NIPT_control_group_separate, file =

paste(ControlGroupDir, "NIPTeR_preliminary_100_control_group.rds", sep=""))

To optimally assess the variabilty and quality of the samples in the control group, variation reduction has to be performed first. In our case, we use a bin GC correction and chi-squared-based variation reduction (χ^2^VR), to create optimal results.

First, the bin GC correction is performed:

> bingc_control_group <- gc_correct(nipt_object = NIPT_control_group_separate,

method = "bin")

To perform a χ^2^VR for the control group, we have to enter an NIPT sample in addition to the control group to the chi_correct formula. Therefore, we take our sample of interest (as used later in this report), create bin counts and perform a bin GC correction on this sample as well.

> raw_sample_bam <- "/PATH/TO/FOLDER/Trisomy21.bam"

> NIPT_raw_sample_bin <- bin_bam_sample( bam_filepath = raw_sample_bam,

separate_strands = T )

> bingc_sample <- gc_correct(nipt_object = NIPT_raw_sample_bin, method = "bin")

Now the χ^2^VR can be performed on the preliminary control group:

> NIPT_bin_chi_corrected_data <- chi_correct( nipt_sample = bingc_sample,

nipt_control_group = bingc_control_group

> NIPT_bin_chi_corrected_controls <- NIPT_bin_chi_corrected_data$control_group

## Diagnose preliminary control group

The variability of the chromosomes of the samples in the reference group are checked using

> table(diagnose_control_group(NIPT_bin_chi_corrected_controls)$

abberant_scores$Sample_name)

This gives the following output:

| Control10 | 1 |
| --- | --- |
| Control11 | 1 |
| Control27 | 1 |
| Control49 | 1 |
| Control51 | 7 |
| Control58 | 1 |
| Control59 | 1 |
| Control65 | 1 |
| Control88 | 6 |
| Control89 | 10 |
| Control90 | 6 |
| Control98 | 1 |

Here, we see that for 12 of the 100 samples at least one of the 44 autosomal chromosome strands shows a standard Z-score that falls outside the 99.7% confidence interval created based on our control group. Eight of these samples only show an aberrant Z-score for one chromosome strand. These are not expected to represent a trisomy, because for a real aberration we expect a positive result for both the forward and the reverse strand. Control samples 51, 88, 89 and 90 all show multiple aberrant scores, indicating there is much more variability in these samples compared to the other 96 control samples. We therefore remove them from the control group.

## Create corrected cleaned control group

Here we remove the 4 aberrant control samples, leaving 96 samples in the control group.

> tmp_controlgroup <- remove_sample_controlgroup("Control51", nipt_control_group = NIPT_control_group_separate)

> tmp_controlgroup <- remove_sample_controlgroup("Control88", nipt_control_group = tmp_controlgroup)

> tmp_controlgroup <- remove_sample_controlgroup("Control89", nipt_control_group = tmp_controlgroup)

> NIPT_control_group_separate_cleaned <- remove_sample_controlgroup("Control90", nipt_control_group =

tmp_controlgroup)

## Diagnose corrected cleaned control group

Now that the aberrant control samples have been removed, we can look at the control group statistics, after doing a (bin) GC correction and χ^2^VR.

> bingc_control_group <- gc_correct(nipt_object =

NIPT_control_group_separate_cleaned, method = "bin")

> NIPT_bin_chi_corrected_data <- chi_correct( nipt_sample = bingc_sample,

nipt_control_group = bingc_control_group

> NIPT_bin_chi_corrected_controls <- NIPT_bin_chi_corrected_data$control_group

> table(diagnose_control_group(NIPT_bin_chi_corrected_controls)$

abberant_scores$Sample_name)

| Control10 | 1 |
| --- | --- |
| Control11 | 1 |
| Control27 | 1 |
| Control32 | 1 |
| Control49 | 1 |
| Control55 | 1 |
| Control58 | 1 |
| Control59 | 1 |
| Control65 | 1 |
| Control85 | 1 |
| Control86 | 1 |
| Control87 | 5 |
| Control91 | 3 |
| Control92 | 3 |
| Control95 | 1 |
| Control97 | 1 |
| Control98 | 4 |

Now four more samples pop up: 87, 91, 92 and 98. For the reasons described above, we now also remove those samples to generate cleaned control group2

## Create second corrected cleaned control group

Remove the 4 other aberrant samples, leaving 92 samples in the control group.

> tmp_controlgroup <- remove_sample_controlgroup("Control87", nipt_control_group =

NIPT_control_group_separate_cleaned)

> tmp_controlgroup <- remove_sample_controlgroup("Control91", nipt_control_group = tmp_controlgroup)

> tmp_controlgroup <- remove_sample_controlgroup("Control92", nipt_control_group = tmp_controlgroup)

> NIPT_control_group_separate_cleaned2 <- remove_sample_controlgroup("Control98", nipt_control_group =

tmp_controlgroup)

## Diagnose second corrected cleaned control group

Now that the aberrant control samples have been removed, we can look at the control group statistics after doing a (bin) GC correction and χ^2^VR.

> bingc_control_group <- gc_correct(nipt_object =

NIPT_control_group_separate_cleaned2, method = "bin")

> NIPT_bin_chi_corrected_data <- chi_correct( nipt_sample = bingc_sample,

nipt_control_group = bingc_control_group

> NIPT_bin_chi_corrected_controls <- NIPT_bin_chi_corrected_data$control_group

> table(diagnose_control_group(NIPT_bin_chi_corrected_controls)$

abberant_scores$Sample_name)

| Control10 | 2 |
| --- | --- |
| Control11 | 1 |
| Control27 | 1 |
| Control32 | 1 |
| Control49 | 1 |
| Control55 | 1 |
| Control58 | 1 |
| Control59 | 1 |
| Control65 | 1 |
| Control85 | 8 |
| Control86 | 2 |
| Control95 | 6 |
| Control97 | 6 |

Now five more samples pop up: 10, 85, 86 and 95 and 97. For the reasons described above, we remove those samples and form cleaned control group3

## Create third corrected cleaned control group

Remove the 5 other aberrant samples, leaving 87 samples in the control group.

> tmp_controlgroup <- remove_sample_controlgroup("Control10$", nipt_control_group =

NIPT_control_group_separate_cleaned2)

> tmp_controlgroup <- remove_sample_controlgroup("Control85", nipt_control_group = tmp_controlgroup)

> tmp_controlgroup <- remove_sample_controlgroup("Control86", nipt_control_group = tmp_controlgroup)

> tmp_controlgroup <- remove_sample_controlgroup("Control95", nipt_control_group = tmp_controlgroup)

> NIPT_control_group_separate_cleaned3 <- remove_sample_controlgroup("Control97", nipt_control_group =

tmp_controlgroup)

## Diagnose third corrected cleaned control group

Now that the aberrant control samples have been removed, we can look at the control group statistics after doing a bin GC correction and χ^2^VR.

> bingc_control_group <- gc_correct(nipt_object =

NIPT_control_group_separate_cleaned3, method = "bin")

> NIPT_bin_chi_corrected_data <- chi_correct( nipt_sample = bingc_sample,

nipt_control_group = bingc_control_group

> NIPT_bin_chi_corrected_controls <- NIPT_bin_chi_corrected_data$control_group

> table(diagnose_control_group(NIPT_bin_chi_corrected_controls)$

abberant_scores$Sample_name)

| Control11 | 1 |
| --- | --- |
| Control12 | 1 |
| Control27 | 1 |
| Control32 | 1 |
| Control36 | 1 |
| Control49 | 1 |
| Control52 | 1 |
| Control55 | 1 |
| Control58 | 1 |
| Control59 | 1 |
| Control65 | 1 |
| Control66 | 1 |
| Control73 | 1 |
| Control83 | 1 |

Here, we see that no sample shows multiple abnormal Z-scores in the third cleaned control group. In all cases only one chromosome strand in a single sample returns a Z-score outside the range -3 to 3. While arguments can be made for removal of these samples from the control group, we recommend to leave these samples in to prevent overfitting the control group.

Interestingly, 11 of the 13 samples now left out of the control group were processed using a different method (they were produced using plasma that was centrifuged once instead of twice: see Johansson and de Boer et al. (2017) for details). No samples produced using single centrifuged plasma are left in the control group.

The third corrected cleaned control group will now be used as the final control group.

Note that a GC-corrected control group can be saved as an rds file and loaded for sample analysis. This can save a lot of time, especially when the LOESS GC correction is used.

## Save cleaned control group for later use

> saveRDS(object = NIPT_control_group_separate_cleaned3, file =

paste(ControlGroupDir, "NIPTeR_cleaned_87_control_group.rds", sep=""))

# Set control group Match QC threshold.

To enable comparison of a sample with the control group a Match QC threshold can be calculated. Because we will use the bin GC and chi corrected ”NIPT_control_group_separate_cleaned3”, the threshold for the Match score is set using the control group that has undergone the same variation reduction methods. The Match QC threshold uses the sum-of-squares of the differences in chromosomal fractions as a metric. The QC threshold is set three standard deviations above the average mean match score.

> match_controls <- list()

> match_score_boundaries <- for (i in

1:length(NIPT_bin_chi_corrected_controls$samples)){

match_list <-

match_control_group(nipt_sample=NIPT_bin_chi_corrected_controls$samples[[i]],

nipt_control_group=NIPT_bin_chi_corrected_controls, n_of_samples=87, mode =

"report")

mean_match <- mean(as.numeric(match_list))

match_controls[[i]] <- mean_match

}

> average_mean_match_controls <- mean(as.numeric(match_controls))

> sd_match <- sd(as.numeric(match_controls))

> QC_threshold <- average_mean_match_controls + 3 * sd_match

The control group Match QC threshold (average mean match score of all control group samples + 3 standard deviations): 5.986 * 10^-7^ with mean 3.598 * 10^-7^.

# Sample analysis

Now that the control group has been created and cleaned the sample of interest can be analyzed and trisomy predictions can be performed. In this example we are going to assume that the cleaned control group has been saved and needs to be loaded first.

The test sample and the 87 sample control group (.rds) can be downloaded from: <https://github.com/molgenis/NIPTeR>.

NIPT_87_control_group <- readRDS( file =

"/PATH/TO/FOLDER/NIPTeR_cleaned_87_control_group.rds" )

Followed by a bin_gc correction:

> bingc_control_group <- gc_correct(nipt_object =

NIPT_87_control_group, method = "bin")

## GC correction and chi-squared-based variation reduction (χ^2^VR)

The lowest variability between samples is achieved by first performing a GC correction, followed by a χ^2^VR. Because a bin GC correction was performed on the control group, the same correction method has to be used on the sample. However, it is also possible to use a LOESS GC correction instead of the bin GC correction on both the sample and the control group.

> raw_sample_bam <- "/PATH/TO/FOLDER/Trisomy21.bam"

> NIPT_raw_sample_bin <- bin_bam_sample( bam_filepath = raw_sample_bam,

separate_strands = T )

OR:

> NIPT_raw_sample_bin <- readRDS( file =

"/PATH/TO/FOLDER/Trisomy21.rds" )

> bingc_sample <- gc_correct(nipt_object = NIPT_raw_sample_bin, method = "bin")

Now the χ^2^VR can be performed. Each sample needs to be corrected based on a control group. In our case, this is the bin GC corrected final control group. After correction both sample and control group are extracted.

> NIPT_bin_chi_corrected_data <- chi_correct( nipt_sample = bingc_sample, nipt_control_group = bingc_control_group )

> NIPT_bin_chi_corrected_sample <- NIPT_bin_chi_corrected_data$sample

> NIPT_bin_chi_corrected_controls <- NIPT_bin_chi_corrected_data$control_group

## Sample Match QC

Now that the sample has been corrected in the same way as the control samples, we can calculate if the match score falls within the range of the control samples.

> mean_match_sample <- mean(as.numeric(match_control_group(nipt_sample=NIPT_bin_chi_corrected_sample, nipt_control_group=NIPT_bin_chi_corrected_controls, n_of_samples=87, mode = "report")))

The sample mean match score (mean of match scores to all control samples) is 3.615 * 10^-7^. This number falls below the Match QC threshold, therefore there is no indication that the control group is not representative for the sample tested.

## Trisomy prediction

The most robust trisomy prediction is done if the same result is calculated using different algorithms (standard Z-score, Normalized Chromosome Value (NCV) and RBZ). If the different prediction methods are in agreement, the risk of a false positive result is lower. However, the sensitivity of the three methods can differ and result in a positive result for one or two methods and a negative result for the other(s). The NCV and RBZ predictions use a subset of chromosomes to predict the number of reads expected to map on the chromosome of interest in case no trisomy is present. In general, these methods have a higher sensitivity than the standard Z-score prediction. However, they are more sensitive to bias. For instance, a maternal copy number variation in one of the chromosomes used as predictor can result in a false positive result. Therefore, to make this bias visible, for both the NCV and RBZ, we recommend performing more than one prediction using different predictor chromosomes to create the models. In case of conflicting results between models the cause of a positive result may not be a trisomy of the chromosome of interest, but rather an issue with one of the predictor chromosomes.

### Standard Z-score

The standard Z-score uses the fraction of reads mapped to the chromosome of interest compared with reads mapped to all other autosomes. In this example we will calculate the Z-scores for chromosomes 13, 18 and 21, which are involved in Patau syndrome, Edwards’ syndrome and Down syndrome, respectively.

To calculate these Z-scores, the same function has to be called three times, each focusing on a different chromosome.

> z_score_13 <- calculate_z_score(nipt_sample = NIPT_bin_chi_corrected_sample, nipt_control_group =

NIPT_bin_chi_corrected_controls, chromo_focus = 13)

> z_score_18 <- calculate_z_score(nipt_sample = NIPT_bin_chi_corrected_sample, nipt_control_group =

NIPT_bin_chi_corrected_controls, chromo_focus = 18)

> z_score_21 <- calculate_z_score(nipt_sample = NIPT_bin_chi_corrected_sample, nipt_control_group =

NIPT_bin_chi_corrected_controls, chromo_focus = 21)

The most important output value is the sample_Zscore, here -1.454 for chromosome 13, -1.207 for chromosome 18 and 8.889 for chromosome 21.

These values can be seen selecting the sample_Zscore field (here shown for chromosome 13).

> Z_score_13$sample_Zscore

A Z-score higher than 3 indicates a trisomy. Thus, here a trisomy 21 is indicated by the standard Z-score.

An important quality metric is the CV of the control groups, which can be calculated by dividing the standard deviation (sd) of reads mapped onto the chromosome of interest on the control samples by the mean of those values (here shown for chromosome 13).

> as.numeric(as.vector(z_score_13$control_group_statistics[2]/

z_score_13$control_group_statistics[1]))*100

The Standard Z-score %CVs are 0.21, 0.30 and 0.36 for chromosomes 13, 18 and 21, respectively. A lower CV indicates a higher sensitivity.

### Normalized Chromosome Value (NCV)

The second type of trisomy prediction included in *NIPTeR* is the NCV. In this method, the set of chromosomes that best predict the number of reads expected to map on the chromosome of interest are selected based on the control group. Using a higher number of denominators (maximum number of chromosomes used to predict the expected number of reads on the chromosome of interest) will mean a longer processing time. For each chromosome of interest, a different template is created. On default the function makes use of a train and a test set. This can lead to a slightly different set of predictors, coefficient of variation (CV) and sensitivity between calculations.

In our example we will use a maximum number of denominators of nine. We will not use a train and test set for NCV template creation here, so that results can be replicated. However, in practice, we recommend using a train and test set when sufficient control samples are available.

> ncv_template_13 <- prepare_ncv(nipt_control_group =

NIPT_bin_chi_corrected_controls, chr_focus = 13, max_elements = 9,

use_test_train_set = F)

> ncv_template_18 <- prepare_ncv(nipt_control_group =

NIPT_bin_chi_corrected_controls, chr_focus = 18, max_elements = 9,

use_test_train_set = F)

> ncv_template_21 <- prepare_ncv(nipt_control_group =

NIPT_bin_chi_corrected_controls, chr_focus = 21, max_elements = 9,

use_test_train_set = F)

Now that the templates have been created, the NCVs can be calculated.

> ncv_score_13 <- calculate_ncv_score(nipt_sample = NIPT_bin_chi_corrected_sample,

ncv_template = ncv_template_13)

> ncv_score_18 <- calculate_ncv_score(nipt_sample = NIPT_bin_chi_corrected_sample,

ncv_template = ncv_template_18)

> ncv_score_21 <- calculate_ncv_score(nipt_sample = NIPT_bin_chi_corrected_sample,

ncv_template = ncv_template_21)

The most important output value is the sample_score, here -1.945 for chromosome 13, -1.208 for chromosome 18 and 9.348 for chromosome 21.

The results of the NCV predictions can be seen using the sample_score field (here for chromosome 13).

> ncv_score_13$sample_score

An NCV higher than 3 indicates a trisomy. Thus, here a trisomy 21 is indicated by the NCV.

An important quality metric is the CV of the control groups, which can be calculated by dividing the standard deviation (sd) of reads mapped on the chromosome of interest on the control samples by the mean of those values (here shown for chromosome 13).

> as.numeric(as.vector(ncv_template_13$control_group_statistics[2]/

ncv_template_13$control_group_statistics[1]))*100

The NCV %CVs are 0.19, 0.26 and 0.34 for chromosomes 13, 18 and 21, respectively. A lower CV indicates a higher sensitivity.

A third type of information that can be useful for quality control are the denominators used in the prediction model (here shown for chromosome 13). If discrepancies between prediction models are seen, knowing which chromosomes are used as internal reference can help identify possible causes of false positive results.

> ncv_score_13$denominators

In our case study the denominators selected for chromosome 13 are chromosomes 2, 3, 8, 10 and 19; for chromosome 18, chromosomes 4, 8 and 20 are used; and for chromosome 21, chromosomes 2, 10, 14, 15, 19 and 20 are used. If, for instance an unexpected low NCV for chromosomes 18 and 21 is calculated, it may be that it is caused by a maternal copy number variation in chromosome 20, since this chromosome is used in both prediction models. To prevent this pitfall and get more information on possible causes of false positive results, a second NCV calculation can be done using a template with different chromosomes used as denominators.

### Regression-based Z-score (RBZ)

The third method for trisomy prediction that *NIPTeR* offers is the RBZ. This method uses a linear regression model using the most informative combinations of chromosomes to predict the expected number of reads on the chromosome of interest. On default the function makes use of a train and a test set, similar to what is done in calculating the NCV score. However, if the number of control samples is small the whole control group can be used to create the model. The user should realize that setting the use_test_train_set to ‘False’ creates a risk of overfitting the model to the control group. In this example, the complete control group is used to create the prediction models, so that results can be replicated. However, in practice we recommend always using a train and test set if the control group size allows it.

> RBZ_13 <- perform_regression( nipt_sample = NIPT_bin_chi_corrected_sample,

nipt_control_group = NIPT_bin_chi_corrected_controls, use_test_train_set = F,

chromo_focus = 13 )

> RBZ_18 <- perform_regression( nipt_sample = NIPT_bin_chi_corrected_sample,

nipt_control_group = NIPT_bin_chi_corrected_controls, use_test_train_set = F,

chromo_focus = 18 )

> RBZ_21 <- perform_regression( nipt_sample = NIPT_bin_chi_corrected_sample,

nipt_control_group = NIPT_bin_chi_corrected_controls, use_test_train_set = F,

chromo_focus = 21 )

The output of the perform regression method can seem complex at first glance, because all information is presented in a single table.

> RBZ_13$prediction_statistics

                         Prediction_set_1    Prediction_set_2    Prediction_set_3    Prediction_set_4

Z_score_sample          -2.59648801660627   -1.62429672921193   -1.64690304909091   -1.30762713914047

CV                    0.00175936217513966 0.00175936217513966 0.00181424565973259 0.00192049047555765

cv_types                   Theoretical_CV      Theoretical_CV        Practical_CV        Practical_CV

P_value_shapiro         0.152089306718635   0.910502024521038   0.880305626003177   0.624267770490538

Predictor_chromosomes   9R  17F  11F  14F    1F  16F  3F  12F    17R  9F  16R  1R     2F  22F  7F  8F

Mean_test_set            1.00000000003949    1.00000000017203   0.999999999511263    1.00000000034467

CV_train_set          0.00170032551197234 0.00174329040489698 0.00181397727655402 0.00192152105911601

> RBZ_18$prediction_statistics

                         Prediction_set_1    Prediction_set_2    Prediction_set_3    Prediction_set_4

Z_score_sample         -0.944890981808874  -0.238887729437042   -1.41936016347958   -1.36938118671063

CV                    0.00228213014724136 0.00234369140758611 0.00262105911860944 0.00274168482974987

cv_types                     Practical_CV        Practical_CV        Practical_CV        Practical_CV

P_value_shapiro         0.051816852082222   0.484952335647949  0.0898038662394176    0.14620120033169

Predictor_chromosomes     8F  4F  19R  1F    17F  1R  19F  8R    17R  3F  11F  6F    4R  10F  5R  16R

Mean_test_set             1.0000000008872   0.999999999644654    1.00000000091137    1.00000000137299

CV_train_set          0.00227980569707411 0.00234297067995428 0.00262013138162407 0.00273827944162555

> RBZ_21$prediction_statistics

                         Prediction_set_1    Prediction_set_2    Prediction_set_3    Prediction_set_4

Z_score_sample           11.1886612119479    8.67800095395375    9.69873221672776    8.92734790498976

CV                    0.00301310131557292 0.00316421702206914 0.00332089678013953 0.00342464372368304

cv_types                     Practical_CV        Practical_CV        Practical_CV        Practical_CV

P_value_shapiro         0.648554528585769   0.084340782326797   0.520772605623114   0.487467120488927

Predictor_chromosomes     4R  3F  16R  1R    10F  4F  22R  3R    2R  1F  14R  20F   10R  12R  5R  11R

Mean_test_set           0.999999998844699    1.00000000071775    1.00000000004629    1.00000000033044

CV_train_set          0.00301574613694026 0.00316961863333957 0.00332479438365028 0.00342949725937507

The prediction statistics show four different prediction models, each showing the RBZ and the control group statistics. The RBZs for chromosome 13 are: -2.596, -1.624, -1.646 and -1.307; for chromosome 18 they are: -0.944, -0.238, -1.419 and -1.369; and for chromosome 21 they are: 11.188, 8.678, 9.698 and 8.927. For each of the chromosomes, the four models are in agreement and either all fall within the -3 to +3 range or all give a Z-score above this range.

The second row shows the CV as compared to 1. To get the %CV, this number can be multiplied by 100 as we have done for the standard Z-score and NCV predictions (here shown for chromosome 13 prediction set 1).

> as.numeric(as.vector(RBZ_13$prediction_statistics[2,1]))*100

This will show the CV 0.001759 as shown above, now represented as %CV 0.1759. A lower CV indicates a higher sensitivity.

The third row in the prediction statistics states if the CV is the real practical CV as calculated using the control group, or a theoretical CV that we use to prevent overfitting of the model. Here, the practical CV is used in all cases, except for the chromosome 13 prediction set 1. This theoretical CV is based on the expected variation if no bias was present, multiplied by the overdispersion rate, which we have set to 15% by default. If the practical CV is lower than the theoretical (minimal possible) CV, the theoretical CV will be used to calculate the Z-score. This will lower the sensitivity of the prediction model slightly, but prevent false positive results. Since the predictors with the strongest correlation are selected for the first prediction model, this model has the highest risk for overfitting. This is also reflected in the increasing CVs going from prediction set 1 to 4. However, when a train and test set is used, the risk of overfitting the models is much smaller.

The fourth row shows the P value of the Shapiro Wilk test performed on the control group. A value below 0.05 indicates that the control group is not normal distributed and that the calculated Z-score should not be used.

The fifth row shows the chromosomes used to create the prediction models. This information can be used to assess discordant results, as discussed in the NCV section above.

The sixth row shows the mean of the test set (or complete set when use_test_train_set = F is used), which should always be close to 1.

The seventh row shows the CV of the train set. This value is identical to the practical CV.

# Conclusion

In our case study, all three prediction models (standard Z-score, NCV and RBZ) return a value between -3 and +3 for chromosomes 13 and 18, indicating that there is no trisomy present (given a sufficient percentage cell-free fetal DNA). The scores for chromosome 21 all show a value above 3. This indicates that a trisomy 21 is present.

# References

Johansson LF, de Boer EN, de Weerd HA, van Dijk F, Elferink MG, Schuring-Blom GH, et al. Novel Algorithms for Improved Sensitivity in Non-Invasive Prenatal Testing. *Sci. Rep*. 2017;7**:**1838
