# Supplementary material for: NIPTeR: an R package for fast and accurate trisomy prediction in non-invasive prenatal testing
Source: BMC Bioinformatics. 2018 Dec 17;19:531. doi: 10.1186/s12859-018-2557-8 (PMC6296037; doi:10.1186/s12859-018-2557-8)
Supplement: Supplementary file 2 — Supplemental information showing the functionality of NIPTeR bin and LOESS GC correction. (DOCX 682 kb) [file 12859_2018_2557_MOESM2_ESM.docx]

# Supplement 2: *NIPTeR* GC correction

The three plots in this supplement show the effect of the LOESS and bin GC correction methods on read counts in the sample we used in the case report (Additional file 1). Figure S2.1 shows the read count for all bins sorted from low to high GC percentage. In this sample, bins with a high GC-percentage have, on average, a higher coverage due to sequencing-specific bias. The fitted LOESS curve shows the overall trend. After performing the LOESS GC-correction (Figure S2.2) or the bin GC-correction (Figure S2.3) the bins having a lower than average read count are corrected upwards and the bins with a higher than average read count are corrected downwards (in this dataset), resulting in a more equally distributed corrected bin read count between bins with different GC-percentages. Because the severity of GC-bias can be different between samples, performing a GC correction can reduce the differences in chromosomal fractions between samples.


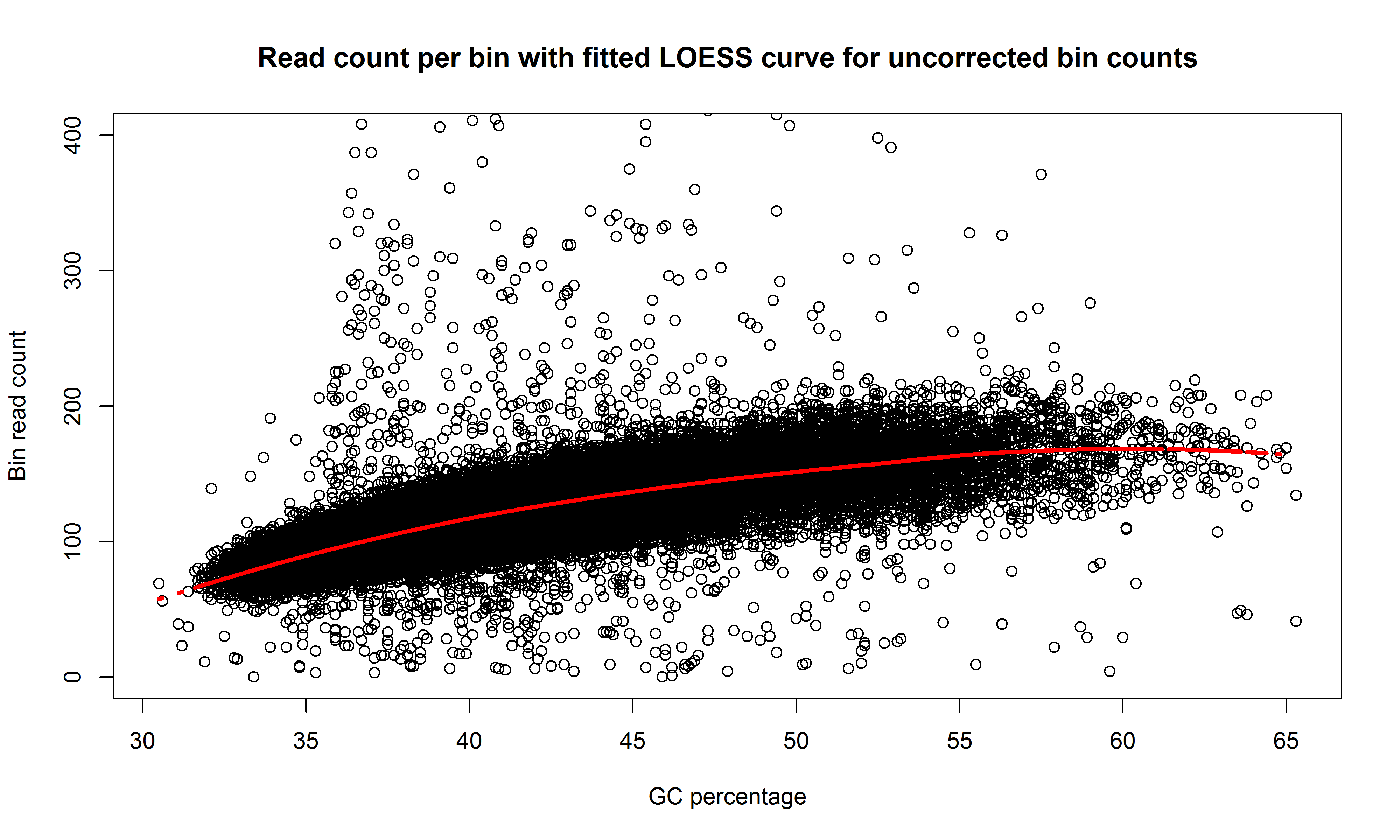


**Figure S2.1 Uncorrected read counts for case report sample with fitted LOESS curve.** Black circles: read count bin. Red line: LOESS fitted line.


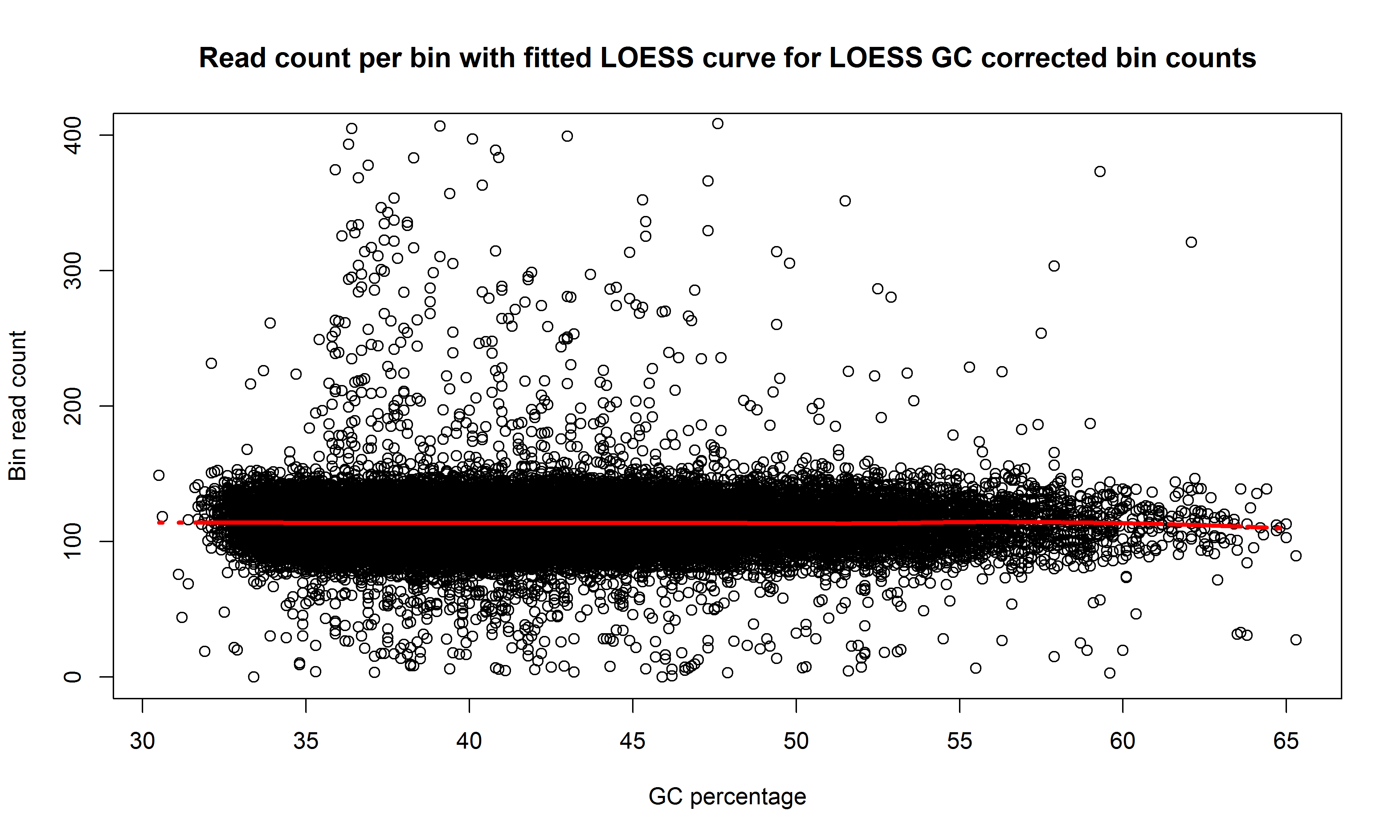


**Figure S2.2 LOESS GC corrected read counts for case report sample with fitted LOESS curve.** Black circles: read count bin. Red line: LOESS fitted line.


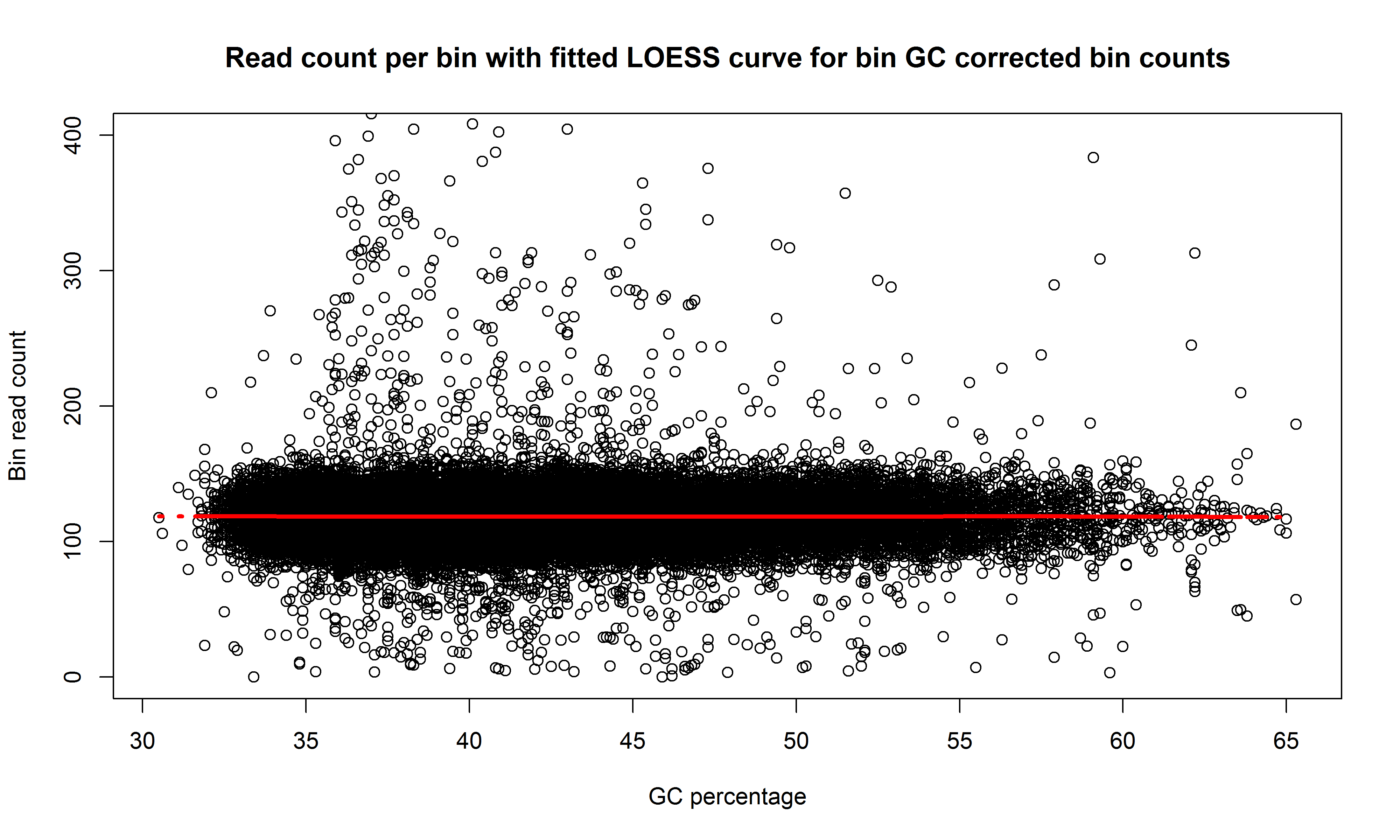


**Figure S2.3 Bin GC corrected read counts for case report sample with fitted LOESS curve.** Black circles: read count bin. Red line: LOESS fitted line.

## Commands to create GC plots

library(NIPTeR)

loess_fit <- function(read_counts){

#Returns a list with all bin count per 0.1% gc interval

all_reads <- sapply(X = indices.gc.percentages.h37, FUN = function(x){(read_counts[x])})

#Get the mean of each interval (for the loess fit)

all_reads_mean <- sapply(X = indices.gc.percentages.h37, FUN = function(x){mean(read_counts[x])})

#2 column table, first column the bin counts, second column corresponding gc percentage

gc_table <- cbind(unlist(all_reads), rep(gc_range, lengths(all_reads)))

#Plot, y-axis 0-400, outliers distort the plot

plot(gc_table[,1] ~ gc_table[,2], ylim=c(0,400),

xlab = "GC percentage", ylab = "Bin read count" )

#Fit loess

loess_fit <- loess(formula =all_reads_mean ~ gc_range, na.action = na.exclude)

#Predict values

loess_pred <- predict(loess_fit)

#Add loess line to plot

lines(gc_range, loess_pred, lwd=3, col= "red")

}

#Gather information

#load sample

NIPT_raw_sample_bin <- readRDS( file = "F:/NIPTeR_validatieset/trisomy_bams/Trisomy04.rds")

sysdata_NIPTeR <- load("F:/NIPTeR_validatieset/sysdata.rda")

gc_percentages_hg37 <- gc_percentages_hg37[1:22,]

gc_percentages_hg37[which(gc_percentages_hg37 < 1)] <- NA

#Get min and max gc percentage (so the range), resolution 0.1%

min_gc <- floor(min(gc_percentages_hg37, na.rm = T) * 10) /10

max_gc <- floor(max(gc_percentages_hg37, na.rm = T)*10) /10

gc_range <- seq(from = min_gc, to = max_gc, by = .1)

#bin correct gc reads

bin_gc_sample <- NIPTeR::gc_correct(nipt_object = NIPT_raw_sample_bin, method = "bin")

#loess correct gc reads

loess_gc_sample <- NIPTeR::gc_correct(nipt_object = NIPT_raw_sample_bin, method = "LOESS")

#Get uncorrected and corrected reads

uncorrected_reads <- NIPT_raw_sample_bin$autosomal_chromosome_reads[[1]]

loess_gc_reads <- loess_gc_sample$autosomal_chromosome_reads[[1]]

bin_gc_reads <- bin_gc_sample$autosomal_chromosome_reads[[1]]

#Create average coverage per 0.1% GC-bin pLots

#uncorrected reads

tiff("F:/NIPTeR_validatieset/Coverage_per_GC_uncorrected_reads.tiff", height = 6, width = 10, units = 'in', res = 400, compression = 'lzw')

loess_fit(uncorrected_reads)

title(main = 'Read count per bin with fitted LOESS curve for uncorrected bin counts')

dev.off()

#LOESS GC corrected reads

tiff("F:/NIPTeR_validatieset/Coverage_per_GC_LOESS_corrected_reads.tiff", height = 6, width = 10, units = 'in', res = 400, compression = 'lzw')

loess_fit(loess_gc_reads)

title(main = 'Read count per bin with fitted LOESS curve for LOESS GC corrected bin counts')

dev.off()

#Bin GC corrected reads

tiff("F:/NIPTeR_validatieset/Coverage_per_GC_bin_corrected_reads.tiff", height = 6, width = 10, units = 'in', res = 400, compression = 'lzw')

loess_fit(bin_gc_reads)

title(main = 'Read count per bin with fitted LOESS curve for bin GC corrected bin counts')

dev.off()
